# Correlation between recurrence of benign paroxysmal positional vertigo and imaging markers of cerebral small vessel disease in middle‑aged and elderly patients

**DOI:** 10.1186/s12883-026-05215-7

**Published:** 2026-08-19

**Authors:** Hui Liu, Jia Liang, Yanan Li, Wei Chen, Min Wan, Yali Gao, Weihua Jia

**Affiliations:** Department of Neurology, Beijing Shijingshan Hospital, Shijingshan Teaching Hospital of Capital Medical University, Beijing, 100043 P. R. China

**Keywords:** Benign paroxysmal positional vertigo, Recurrence, Cerebral small vessel disease, Imaging markers, Vascular risk factors, Middle‑aged and elderly patients

## Abstract

**Background:**

Benign paroxysmal positional vertigo (BPPV) is the most common peripheral vestibular disorder in middle‑aged and elderly individuals, with a high recurrence rate that severely impairs quality of life. Cerebral small vessel disease (CSVD) may contribute to BPPV recurrence by impairing vestibular microcirculation, but the correlation between CSVD imaging markers and BPPV recurrence remains unclear. This study aimed to explore their relationship and identify independent risk factors for BPPV recurrence.

**Methods:**

A retrospective study was conducted including 131 middle‑aged and elderly patients with newly diagnosed BPPV from December 2024 to October 2025. All patients underwent diagnosis and repositioning treatment using the SRM‑IV system and were followed for 4 months. Cranial 3.0T MRI was performed to evaluate CSVD imaging markers according to the 2023 STRIVE‑2 criteria. Clinical and imaging data were compared between recurrence and non‑recurrence groups. Statistical analyses were performed using SPSS 26.0, including t‑test, Mann‑Whitney U test, χ² test, and binary logistic regression.

**Results:**

The 4‑month recurrence rate was 29.0%. The recurrence group had significantly higher prevalence of hypertension, diabetes mellitus, hyperlipidemia, migraine, and higher 24‑hour systolic blood pressure variability (all *P* < 0.05). The recurrence group also showed higher rates of lacunar infarction and recent subcortical small infarcts, as well as more severe white matter hyperintensities and enlarged perivascular spaces (all *P* < 0.05). Logistic regression identified hypertension, diabetes mellitus, hyperlipidemia, migraine, and 24‑hour systolic blood pressure variability as independent risk factors. Enlarged perivascular spaces and lacunar infarction were independent imaging predictors of BPPV recurrence.

**Conclusions:**

Vascular risk factors and CSVD imaging markers, especially enlarged perivascular spaces and lacunar infarction, are closely associated with BPPV recurrence, likely mediated by microcirculatory ischemic injury. Clinical evaluation and individualized intervention of these factors may help reduce recurrence risk and improve prognosis in middle‑aged and elderly patients.

**Trial registration:**

Not applicable (retrospective observational study without health care intervention).

## Introduction

Benign paroxysmal positional vertigo (BPPV) stands as the most common peripheral vestibular disorder, accounting for 17% to 30% of all causes leading to clinical vertigo. Its core clinical manifestations are characterized by sudden and transient vertigo, along with characteristic nystagmus triggered by head - position changes. It follows a self - limiting course yet exhibits a high tendency for recurrence [1]. The middle-aged and elderly population represents high risk group for BPPV. With the accelerating process of population aging, the incidence of BPPV in this group has been increasing year after year. Relevant studies have reported that the recurrence rate of BPPV in middle-aged and elderly patients can range from 20% to 40% [2, 3]. Recurrent episodes not only severely impair patients’ daily living abilities and mental health but also notably elevate the risk of falls, fractures, and disability, imposing a heavy medical and economic burden on families and society [4–6]. Therefore, clarifying the risk factors and objective early warning indicators for BPPV recurrence holds great clinical significance for the precise prevention and control of the disease among the middle-aged and elderly population.

Cerebral small vessel disease (CSVD) is a group of cerebrovascular diseases distinguished by pathological alterations in intracerebral small arteries, arterioles, capillaries, and small veins [7]. The clinical manifestations of CSVD are insidious, and its diagnosis primarily relies on cranial magnetic resonance imaging (MRI) [8, 9]. Typical imaging markers encompass white matter hyperintensities, enlarged perivascular spaces, lacunar infarction, cerebral microbleeds, and recent subcortical small infarcts [10]. Existing studies have confirmed that CSVD may participate in the pathogenesis of BPPV by influencing the blood supply of the vestibular central and peripheral systems [11–13]. There are evident controversies in the current research on the correlation between CSVD imaging markers and BPPV recurrence. Some studies have reported a close association between white matter hyperintensities and BPPV recurrence, but no significant independent correlation between them was confirmed [14, 15].

Meanwhile, the specific correlation between different CSVD imaging markers and BPPV recurrence has not been clearly defined. There is a lack of objective imaging indicators applicable in clinical practice to evaluate the recurrence risk of BPPV, which hardly meets the clinical demand for the precise prevention and control of BPPV.

Based on these research gaps, this study focused on middle-aged and elderly patients newly diagnosed with BPPV. It systematically evaluated the distribution characteristics of various CSVD imaging markers according to the 2023 STRIVE − 2 standards [10] and combined them with clinical vascular risk factors to explore the correlation between these factors and BPPV recurrence. The study aimed to identify the independent risk factors and objective imaging early warning indicators for BPPV recurrence in middle-aged and elderly patients, fill the research gap in the correlation between CSVD and BPPV recurrence, and provide theoretical and practical support for the research on the vascular pathogenesis of BPPV recurrence and the clinical individualized prevention and control of the disease.

## Materials and methods

### Study design and participants

A total of 136 consecutive patients aged ≥ 60 years with newly diagnosed primary BPPV admitted between December 2024 and October 2025 were initially enrolled. Patients who did not complete cranial MRI (*n* = 2) or were lost to follow‑up (*n* = 3) were excluded. Finally, 131 patients were included and divided into non‑recurrence (*n* = 93) and recurrence (*n* = 38) groups based on 4‑month follow‑up outcomes.

### Inclusion criteria

1. Diagnosis of primary BPPV according to the 2017 Clinical Practice Guidelines [4], confirmed by the SRM‑IV system [16, 17];
2. Age ≥60 years;
3. Complete clinical, imaging, and follow‑up data;
4. No contraindications to MRI.

### Exclusion criteria

1. Secondary BPPV;
2. Other organic ear, cranial, or severe large‑vessel diseases;
3. Medications affecting vestibular function, blood pressure, glucose, or coagulation within the previous 3 months;
4. Severe systemic diseases, cognitive or communication disorders;
5. Pregnancy or lactation.

BPPV recurrence was defined as symptom relapse in the same semicircular canal after at least 4 weeks of complete symptom resolution.

### Clinical data collection

Baseline data included age, sex, medical history (hypertension, diabetes mellitus, hyperlipidemia, migraine, tinnitus), and 24‑hour systolic blood pressure variability measured by standard blood pressure monitoring. In this study, 24-hour systolic blood pressure variability was assessed using the standard deviation (SD) method. All valid blood pressure measurements were collected via 24-hour ambulatory blood pressure monitoring, with readings recorded every 30 min during the daytime and every 60 min overnight. The SD value was computed with SPSS 26.0 software. This analytical approach was adopted in accordance with previously published studies [18, 19].

### Cranial MRI and CSVD evaluation

All patients underwent 3.0T cranial MRI within 1 week after diagnosis. CSVD imaging markers were evaluated according to the 2023 STRIVE‑2 criteria:

- White matter hyperintensities: Fazekas scale (0–3 grades) [20];
- Enlarged perivascular spaces: Potter scoring (0–4 grades) [21];
- Lacunar infarction: 3–15 mm cavity with cerebrospinal fluid signal;
- Cerebral microbleeds: detected on susceptibility‑weighted imaging (SWI);
- Recent subcortical small infarcts: hyperintense on diffusion‑weighted imaging (DWI), diameter ≤ 20 mm, onset within 3 weeks.

All images were independently evaluated by a senior radiologist. Intra‑observer consistency was tested in 20 randomly selected patients (Kappa > 0.75).

### Treatment and follow‑up

All patients underwent diagnosis and repositioning treatment using the SRM‑IV system. Patients were followed for 4 months via outpatient review or telephone contact, and recurrence was recorded. The rationale for the 4-month follow-up duration is as follows. Previous studies have indicated that most BPPV recurrences occur within 3–6 months after initial treatment [2, 3]. A 4-month follow-up window can effectively capture recurrent events while balancing follow-up feasibility and data completeness. Our results show a 4-month recurrence rate of 29.0%, which is consistent with the previously reported range of 20%–40% [2, 3], further supporting the rationality and validity of this follow-up timeframe.

### Statistical analysis

SPSS 26.0 was used for statistical analysis. Measurement data conforming to normal distribution were expressed as mean ± standard deviation and compared using independent samples t‑test. Non‑normally distributed data were expressed as median (interquartile range) and compared using Mann‑Whitney U test. Categorical data were expressed as n (%) and compared using χ² test. Ordinal data were analyzed using Mann‑Whitney U test. Variables with *P* < 0.05 in univariate analysis were included in binary logistic regression to identify independent risk factors. A two‑sided *P* < 0.05 was considered statistically significant.

## Results

### Baseline clinical characteristics

The overall 4‑month recurrence rate was 29.0%. There were no significant differences in age, sex, or tinnitus between the two groups (all *P* > 0.05). The recurrence group had significantly higher proportions of hypertension, diabetes mellitus, hyperlipidemia, migraine, and higher 24‑hour systolic blood pressure variability (all *P* < 0.05). The detailed baseline clinical data of the two groups are shown in Table 1.

**Table 1:** Comparison of baseline clinical data between the two groups of middle-aged and elderly BPPV patients

Items	Non-recurrence group (*n* = 93)	Recurrence group (*n* = 38)	*P* value
Male [n(%)]	30(32.3)	13(34.2)	0.829
Age [years, M(Q₁,Q₃)]	68(64.25,73.75)	66.5(63.25,73.25)	0.889
Hypertension history [n(%)]	20(21.5)	16(42.1)	0.017
Diabetes mellitus history [n(%)]	14(15.1)	15(39.5)	0.002
Hyperlipidemia [n(%)]	27(29.0)	18(47.4)	0.011
Migraine [n(%)]	5(5.4)	8(21.1)	0.016
Tinnitus [n(%)]	30(32.3)	18(47.4)	0.103
24-hour systolic blood pressure variability	0.1847 ± 0.08185	0.2771 ± 0.07226	< 0.001

### Comparison of CSVD imaging markers

The recurrence group had significantly higher incidence of lacunar infarction (68.4% vs. 32.3%, *P* < 0.001) and recent subcortical small infarcts (21.1% vs. 6.5%, *P* = 0.032). The recurrence group also showed more severe white matter hyperintensities and enlarged perivascular spaces (both *P* < 0.001). No significant difference was observed in cerebral microbleeds (*P* = 1.000). The detailed comparison results of CSVD unordered variables are shown in Table 2, and the comparison results of CSVD ordered variables are shown in Table 3.

**Table 2:** Comparison of CSVD unordered variables between the two groups

Subgroups	Non-recurrence group (*n* = 93)	Recurrence group (*n* = 38)	χ² value	*P* value
Lacunar infarction [n(%)]	30(32.3)	26(68.4)	14.415	< 0.001
Cerebral microbleeds [n(%)]	11(11.8)	5(13.2)	0.00	1.000
Recent subcortical small infarcts [n(%)]	6(6.5)	8(21.1)	4.593	0.032

**Table 3:** Comparison of CSVD ordered variables between the two groups

Subgroups	Non-recurrence group (*n* = 93)	Recurrence group (*n* = 38)	U value	Z value	*P* value
White matter hyperintensities (Grade 0/1/2/3) [n(%)]	45(48.4)/36(38.7)/10(10.8)/2(2.2)	6(15.8)/7(18.4)/20(52.6)/5(13.2)	778	-5.307	< 0.001
Enlarged perivascular spaces (Grade 0/1/2/3) [n(%)]	54(58.1)/28(30.1)/8(8.6)/3(3.2)	7(18.4)/10(26.3)/15(39.5)/6(15.8)	826	-5.120	< 0.001

### Multivariate logistic regression analysis

Binary logistic regression identified the following as independent risk factors for BPPV recurrence:

- Hypertension (OR = 4.013, 95%CI 1.338–12.033, *P* = 0.013)
- Diabetes mellitus (OR = 4.552, 95%CI 1.492–13.883, *P* = 0.008)
- Hyperlipidemia (OR = 3.675, 95%CI 1.224–11.039, *P* = 0.020)
- Migraine (OR = 4.950, 95%CI 1.054–23.246, *P* = 0.043)
- 24‑hour systolic blood pressure variability (OR = 1.01 × 10⁻⁶, 95%CI 1.33 × 10⁻⁹–0.001, *P* < 0.001)

After adjusting for confounders, two CSVD imaging markers remained independently associated with recurrence:

- Enlarged perivascular spaces (OR = 3.844, 95%CI 1.726–8.563, *P* = 0.001)
- Lacunar infarction (OR = 4.683, 95%CI 1.291–16.990, *P* = 0.019)

White matter hyperintensities, recent subcortical small infarcts, and cerebral microbleeds showed no independent association with BPPV recurrence (all *P* > 0.05).

The detailed parameters of the multivariate Logistic regression analysis of BPPV recurrence and the independent correlation analysis between BPPV recurrence and each CSVD subgroup are shown in Table 4.

**Table 4:** Multivariate logistic regression analysis of BPPV recurrence and its association with CSVD subgroups in middle-aged and elderly patients

Factors/ Subgroups	B value	S.E value	OR value	95%CI	*P* value
Hypertension	1.39	0.56	4.013	1.338 ~ 12.033	0.013
Diabetes mellitus	1.516	0.569	4.552	1.492 ~ 13.883	0.008
Hyperlipidemia	1.302	0.561	3.675	1.224 ~ 11.039	0.02
Migraine	1.599	0.789	4.95	1.054 ~ 23.246	0.043
24-hour systolic blood pressure variability	-13.803	3.385	1.01E-06	1.33E-09 ~ 0.001	< 0.001
Enlarged perivascular spaces	1.347	0.409	3.844	1.726 ~ 8.563	0.001
Lacunar infarction	1.544	0.657	4.683	1.291 ~ 16.990	0.019
White matter hyperintensities	0.669	0.387	1.953	0.915 ~ 4.168	0.084
recent subcortical small infarcts	0.926	0.518	2.525	0.987 ~ 6.462	0.053

## Discussion

This retrospective study investigated the correlation between CSVD imaging markers and BPPV recurrence in 131 middle‑aged and elderly patients, which is consistent with the design of previous retrospective studies focusing on BPPV recurrence [12, 13]. The 4‑month recurrence rate was 29.0%. We confirmed that hypertension, diabetes mellitus, hyperlipidemia, migraine are independent clinical risk factors, which is in line with the conclusions of relevant studies on elderly BPPV patients [22, 23]. Importantly, enlarged perivascular spaces and lacunar infarction were independent imaging predictors of BPPV recurrence. These findings support that microcirculatory ischemic injury related to vascular risk factors [24, 25] and CSVD contributes to BPPV recurrence [12, 13].

The pathogenesis of BPPV recurrence is closely related to otolith instability and vestibular dysfunction. The vestibular system relies on the vertebrobasilar circulation and is highly sensitive to ischemia. CSVD and traditional vascular risk factors may impair small vessel perfusion, disrupt otolith metabolism, and damage vestibular pathways, thereby increasing recurrence risk. which is consistent with the pathological mechanism of BPPV recurrence proposed in recent studies [26–28].

Our results are consistent with previous studies showing that hypertension, diabetes, hyperlipidemia, and migraine are associated with BPPV recurrence. These conditions cause endothelial dysfunction, arteriosclerosis, and microvascular hypoperfusion, compromising vestibular blood supply [22, 23, 29].

Notably, this study first identified 24‑hour systolic blood pressure variability as an independent risk factor for BPPV recurrence, which supplements the findings of existing studies on 24-hour blood pressure variability and BPPV recurrence [18, 19]. Excessive blood pressure fluctuation may reflect more severe vascular damage than sustained hypertension and represents a valuable target for clinical monitoring and intervention, which is supported by studies on blood pressure variability and cerebral small vessel damage [18, 19, 30, 31].

For CSVD markers, enlarged perivascular spaces and lacunar infarction were independently associated with recurrence. Enlarged perivascular spaces reflect early small vessel damage and increased vascular permeability, representing a promising early imaging marker, which is consistent with the role of perivascular spaces in CSVD evaluation [32–35]. Lacunar infarction indicates overt small vessel occlusion and may disrupt vestibular signal transmission, and similar conclusions have been drawn in studies on lacunar infarction and BPPV recurrence [12, 13].

White matter hyperintensities were more severe in the recurrence group but showed no independent predictive value after adjustment, possibly because they represent chronic ischemic changes whose effects are obscured by stronger vascular risk factors, which is consistent with the controversial findings on white matter hyperintensities and BPPV recurrence in previous studies [13, 15]. Recent subcortical small infarcts and cerebral microbleeds were not independently associated with recurrence, suggesting that BPPV recurrence is more closely related to chronic ischemic small vessel disease rather than acute infarction or micro‑hemorrhage, which is different from the potential association between cerebral microbleeds and CSVD reported in some studies [36, 37].

The strengths of this study include systematic evaluation of CSVD using the 2023 STRIVE‑2 criteria, first report of the predictive value of 24‑hour systolic blood pressure variability, and clear identification of independent CSVD imaging markers. Detailed limitations of this study and future research directions have been summarized separately after the Conclusions section.

## Conclusions

Hypertension, diabetes mellitus, hyperlipidemia, migraine, and 24‑hour systolic blood pressure variability are independent vascular risk factors for BPPV recurrence in middle‑aged and elderly patients. Enlarged perivascular spaces and lacunar infarction, as typical CSVD imaging markers, can serve as objective imaging indicators for predicting BPPV recurrence.

Microcirculatory ischemic injury caused by vascular risk factors and CSVD is an important pathological basis for recurrent BPPV. Clinical practice should emphasize comprehensive evaluation of vascular risk factors and CSVD imaging markers, and implement individualized interventions to reduce recurrence and improve prognosis.

Nevertheless, this study has several limitations. First, this was a single-center retrospective observational study with a total sample of only 131 elderly patients with newly diagnosed BPPV. Given the high overall incidence of BPPV in middle-aged and elderly populations, the small sample size and single-hospital recruitment may restrict the generalizability of our findings to patients from different medical institutions or regions. Second, the 4-month follow-up period is relatively short to assess long-term BPPV recurrence outcomes. Third, this study did not incorporate serological vascular biomarkers for joint analysis. Fourth, subgroup analysis based on different lesion types of semicircular canals was not performed. Hence, multi-center prospective studies with larger sample sizes and longer follow-up duration are warranted to verify our findings.

## Data Availability

The datasets used and analyzed during the current study are available from the corresponding author on reasonable request, with approval from the Ethics Committee of Beijing Shijingshan Hospital.

## Abbreviations

BPPV: Benign paroxysmal positional vertigo
CSVD: Cerebral small vessel disease
MRI: Magnetic resonance imaging
OR: Odds ratio
CI: Confidence interval
SWI: Susceptibility‑weighted imaging
DWI: Diffusion‑weighted imaging
